# Supplementary material for: Rapid Identification of Paeoniae Radix and Moutan Radicis Cortex Using a SCAR Marker-Based Conventional PCR Assay
Source: Plants (Basel). 2022 Oct 27;11(21):2870. doi: 10.3390/plants11212870 (PMC9653921; doi:10.3390/plants11212870)
Supplement: Supplementary file 1 [file plants-11-02870-s001.zip › plants-1905463-supplementary.pdf]

Table S1. List of 35 *Paeonia* accessions examined in this study.

Plant species	Sample ID	Voucher no.	GenBank accession no.	
			rDNA-ITS	*rbc*L
Paeonia lactiflora Pall.	PL JS	KIOM2013KR02-50	KT944679	KT944713
	PL GC	KIOM2013KR04-45	KT944680	KT944714
	PL HC	KIOM2013KR05-07	KT944681	KT944715
	PL HL	KIOM2013CN01-21	KT944682	KT944716
	PL YG	KIOM2013CN01-46	KT944683	KT944717
	PL TY	KIOM2012KR03-05	OP106991	OP253111
	PL AS	KIOM2013KR03-01	OP106992	OP253112
	PL JJ	KIOM2015KR01-38	OP106993	OP253113
	PL SJ	KIOM2015KR02-47	OP106994	OP253114
	PL GL	KIOM2013CN01-27	OP106995	OP253115
Paeonia japonica (Makino) Miyabe & Takeda	PJ GR	KIOM2007KR01-02	KT944684	KT944718
	PJ MJ	KIOM2007KR01-03	KT944685	KT944719
	PJ IJ	KIOM2007KR02-13	KT944686	KT944720
	PJ HY	KIOM2012KR07-04	KT944687	KT944721
	PJ SGP	KIOM2013KR03-47	KT944688	KT944722
	PJ AD	KIOM2015KR01-27	OP106996	OP253116
	PJ GC	KIOM2015KR02-32	OP106997	OP253117
	PJ JS	KIOM2013KR03-04	OP106998	OP253118
	PJ SC	KIOM2013KR04-11	OP106999	OP253119
Paeonia anomala subsp. *veitchii* (Lynch) D.Y.Hong & K.Y.Pan	PA SC-1	KIOM2012CN02-10	KT944689	KT944723
	PA SC-2	KIOM2012CN02-11	KT944690	KT944724
	PA SC-3	KIOM2012CN02-12	KT944691	KT944725
	PA SC-4	KIOM2012CN02-13	KT944692	KT944726
	PA SC-5	KIOM2012CN02-14	OP107000	OP253120
	PA SC-6	KIOM2012CN02-15	OP107001	OP253121
	PA GS	KIOM2007GN02-08	OP107002	OP253122
Paeonia suffruticosa	PS AD-1	KIOM2013KR13-49	KT944693	KT944727
	PS AD-2	KIOM2013KR13-50	KT944694	KT944728
	PS AD-3	KIOM2014KR01-13	KT944695	KT944729
	PS JJ-1	KIOM2015KR01-23	OP107003	OP253123
	PS JJ-2	KIOM2015KR01-24	OP107004	OP253124
	PS JJ-3	KIOM2015KR01-25	OP107005	OP253125
	PS JJ-4	KIOM2015KR01-26	OP107006	OP253126

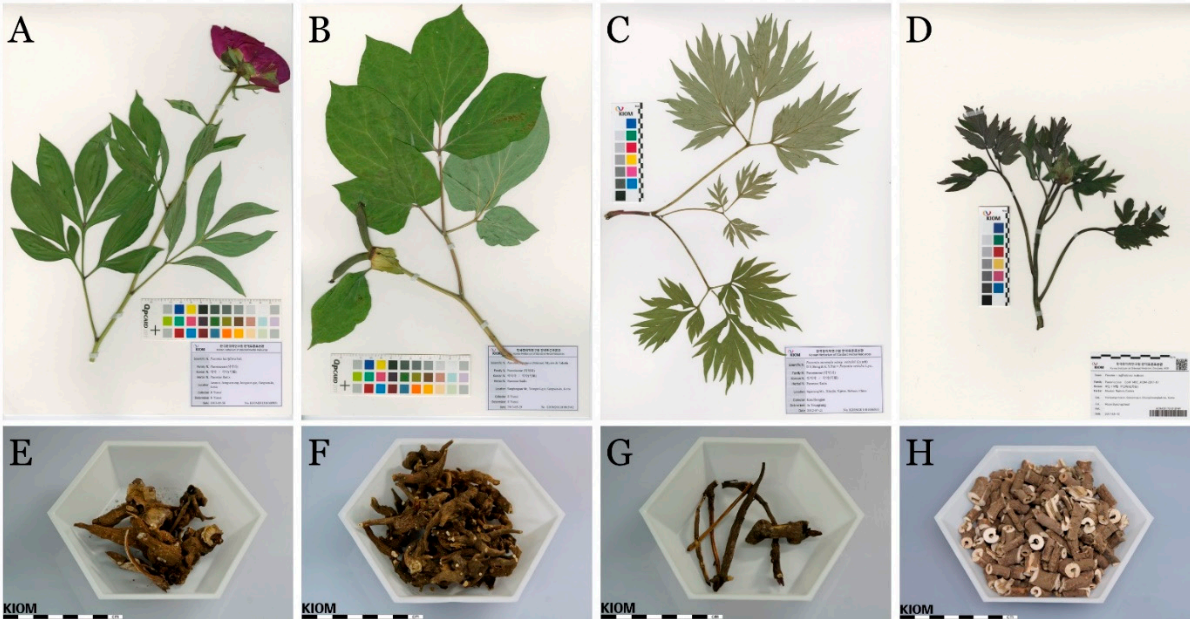

Figure S1. Specimens (A, B, C, D) of *Paeonia* species and photos (E, F, G, H) of herbal medicines. *P. lactiflora* (A, E); *P. japonica* (B, F); *P. anomala* subsp. *veitchii* (C, G); *P. suffruticosa* (D, H).

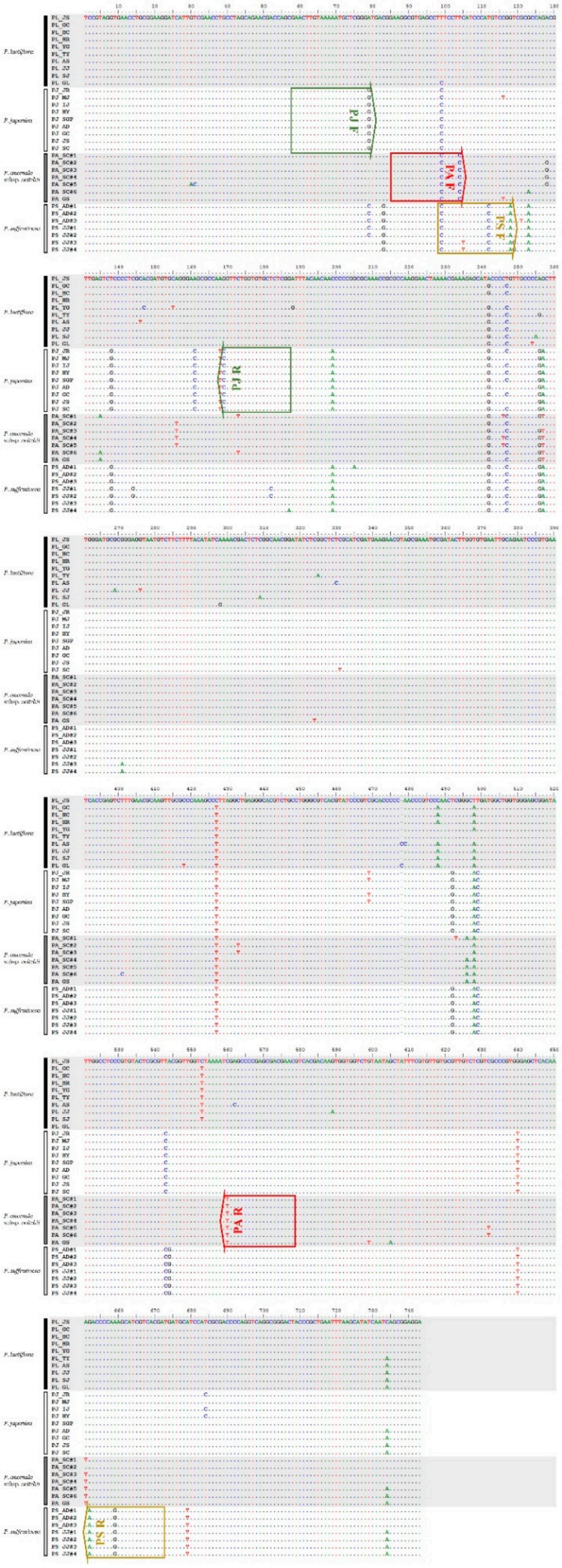

Figure S2. Multiple sequence alignment of the internal transcribed spacer (ITS) region of 35 *Paeonia* accessions. The alignment was constructed using ClustalW. Arrows indicate the binding sites of the species-specific SCAR primers designed in this study.

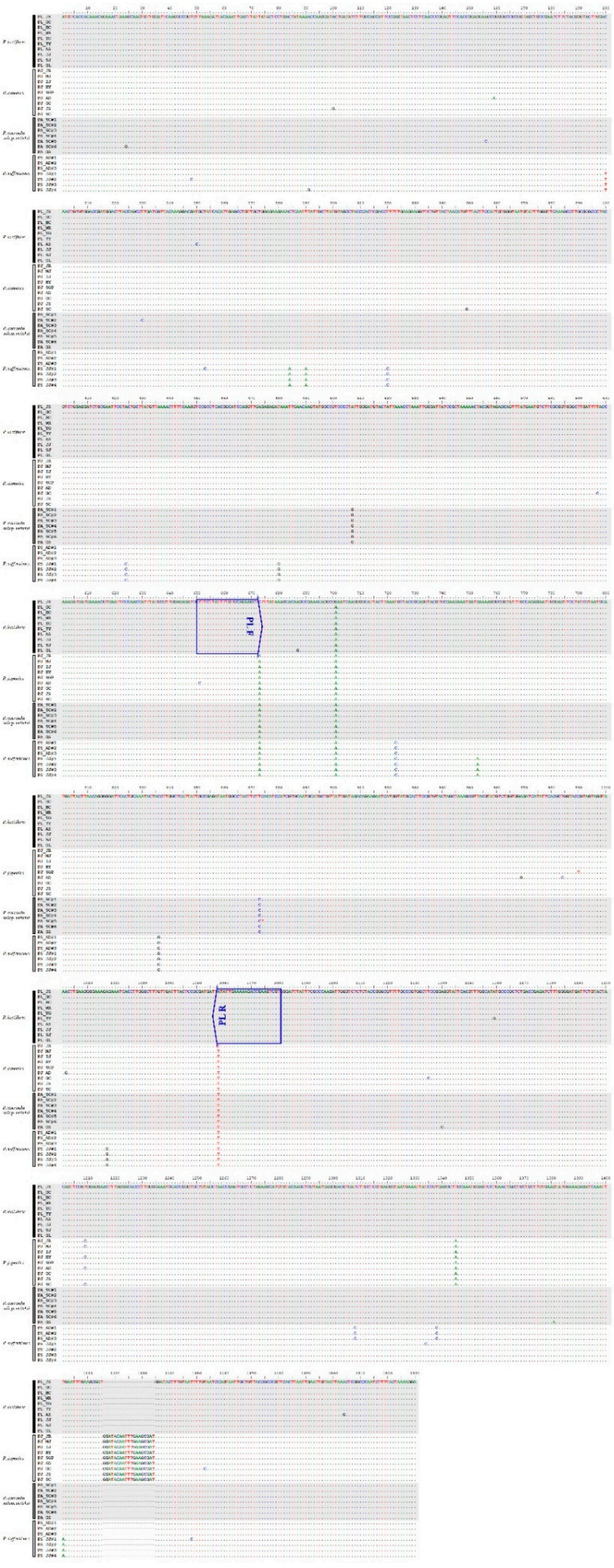

Figure S3. Multiple sequence alignment of the *rbc*L regions of 35 *Paeonia* accessions. The alignment was constructed using ClustalW. Arrows indicate the binding sites of the species-specific SCAR primers designed in this study.

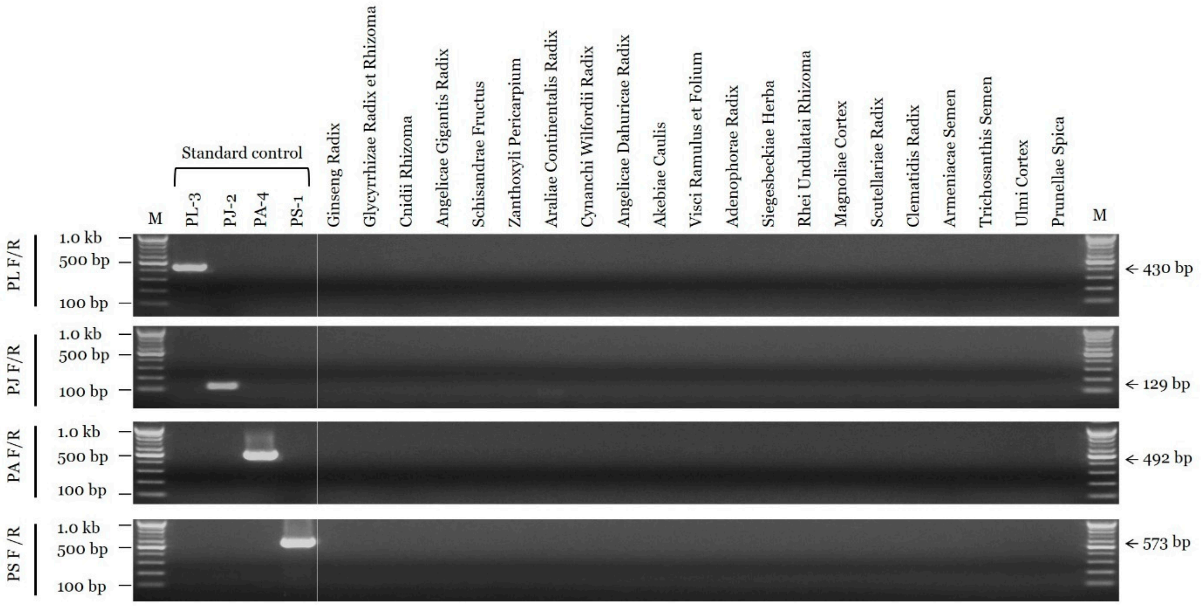

Figure S4. Assessment of the specificity of SCAR primers using 21 herbal medicine-related plant species. SCAR primer pairs (PL-F/-R, PJ-F/-R, PA-F/-R, and PS-F/-R) and 100 bp, 500 bp, and 1.0 kb bands of the 100 bp DNA marker (M) are indicated on the left. Standard controls (PL-3, PJ-2, PA-4, and PS-1) are described in Table S1. Arrows and numbers on the right indicate the amplicon size.

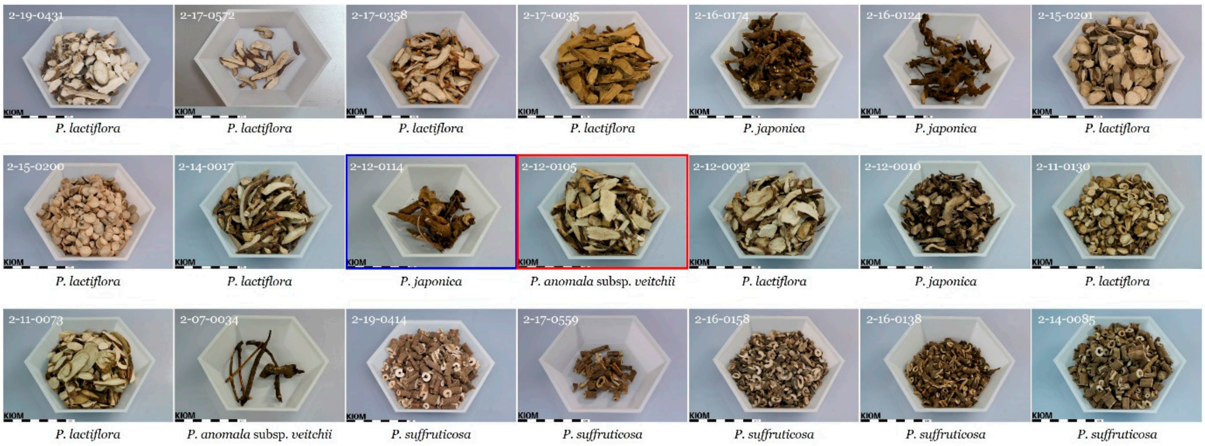

Figure S5. Morphological assay of PR and MRC samples sold in the Korean herbal market. Colored squares indicate that the sample was identified as *P. japonica* (blue) and *P. anomala* subsp. *veitchii* (red) in the morphological assay and identified as *P. lactiflora* in the genetic assays (SCAR and DNA barcoding).

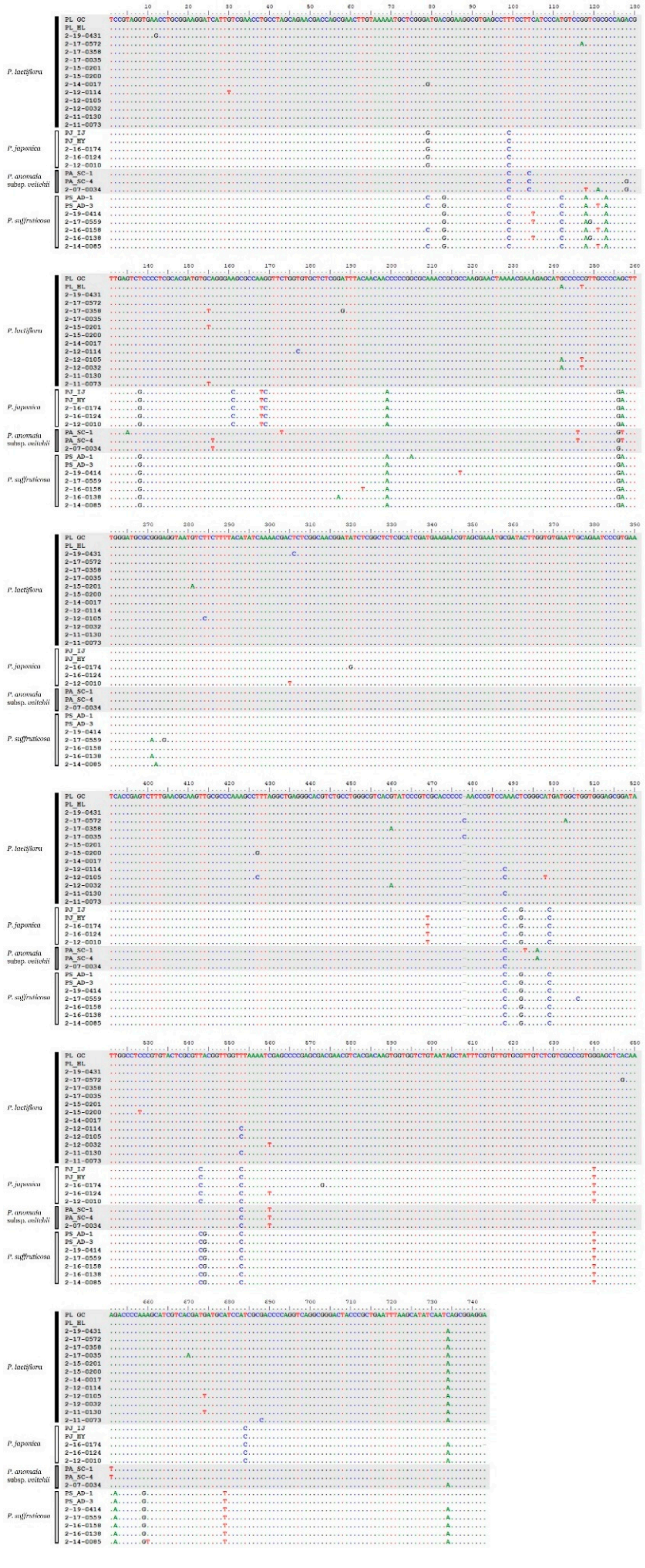

Figure S6. Multiple sequence alignment of the internal transcribed spacer (ITS) region of PR and MRC sold in the Korean herbal market. The alignment was constructed using ClustalW.
